# Endogenous retroviruses in the origins and treatment of cancer

**DOI:** 10.1186/s13059-021-02357-4

**Published:** 2021-05-10

**Authors:** Natasha Jansz, Geoffrey J. Faulkner

**Affiliations:** 1 Mater Research Institute - University of Queensland, TRI Building, Woolloongabba, QLD 4102 Australia; 2 Queensland Brain Institute, University of Queensland, Brisbane, QLD 4072 Australia

## Abstract

Endogenous retroviruses (ERVs) are emerging as promising therapeutic targets in cancer. As remnants of ancient retroviral infections, ERV-derived regulatory elements coordinate expression from gene networks, including those underpinning embryogenesis and immune cell function. ERV activation can promote an interferon response, a phenomenon termed viral mimicry. Although ERV expression is associated with cancer, and provisionally with autoimmune and neurodegenerative diseases, ERV-mediated inflammation is being explored as a way to sensitize tumors to immunotherapy. Here we review ERV co-option in development and innate immunity, the aberrant contribution of ERVs to tumorigenesis, and the wider biomedical potential of therapies directed at ERVs.

**Supplementary Information:**

The online version contains supplementary material available at 10.1186/s13059-021-02357-4.

## Introduction

Endogenous retroviruses (ERVs) are uninvited and collectively indispensable residents in the human genome. Nearly 9% of our DNA is composed of identifiable ERVs [1] (Table 1). Most ERVs entered the germline via retroviral infection of a distant ancestor over 30 million years ago [3] and accumulated mutations at a neutral rate [4]. Some ERV protein-coding sequences have however assumed new and essential roles. The convergent evolution of placental Syncytin proteins is the foremost example of this, while others, such as Arc protein transaction of neuronal synapse plasticity, continue to emerge [5, 6]. More broadly, ERVs are a major source of gene regulatory innovation, as observed in the pluripotent cells of the early embryo [7–12], placental tissues [13–15], the immune system [16–18], and other biological contexts [19–27]. Normal development, and the proper function of many cells and organs, therefore relies upon ERVs and their derivatives.

**Table 1:** Intersection of RepeatMasker defined reference genome (hg38) ERV sequences with ENCODE candidate cis-regulatory elements [2]. Values in brackets represent fold-change compared to random sampling, with bold font highlighting > 50% depletion (italicized) or enrichment (underlined). CTCF-bound elements have predicted insulator or looping function

Family	Reference genome (%)	Promoter (%, ***n*** = 60,338)	Proximal enhancer (%, ***n*** = 141,830)	Distal enhancer (%, ***n*** = 667,599)	CTCF-bound (%, ***n*** = 56,766)
ERV	8.860	8.472 (1.0)	6.124 (0.7)	12.786 (1.4)	17.317 **(2.0)**
LTR5Hs (HERV-K)	0.019	*0.008* ***(0.4)***	0.032 **(1.6)**	0.037 **(1.9)**	0.014 (0.7)
LTR7 (HERV-H)	0.031	0.028 (0.9)	0.054 **(1.7)**	0.118 **(3.9)**	0.037 (1.2)
LTR17 (HERV-W)	0.016	0.017 (1.1)	0.008 (0.5)	0.012 (0.8)	0.012 (0.8)

**Fig. 1:**
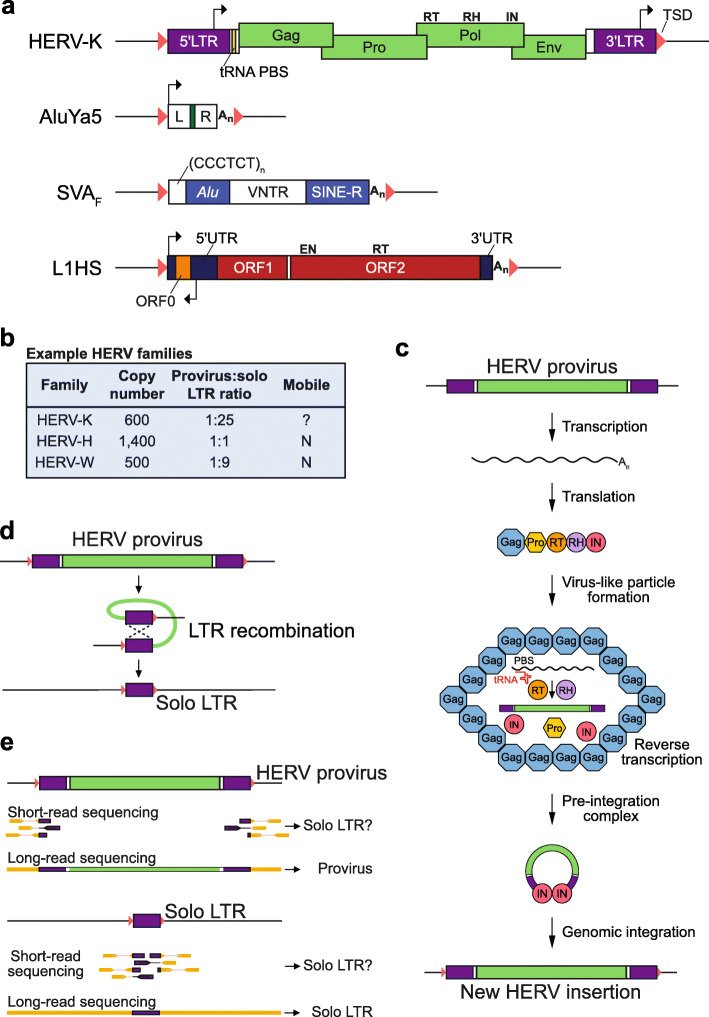
Human transposable elements. **a** HERV-K, AluYa5, SVA_F_ and L1HS (L1 human-specific) are the youngest human ERV, *Alu*, SVA, and L1 families, respectively. New retrotransposition events are flanked by hallmark target site duplications (TSDs, pink triangles). HERV-K is ~9 kbp in length, contains a tRNA reverse transcription primer binding site (PBS), and encodes overlapping *Gag*, *Pro*, *Pol*, and *Env* ORFs. Amongst other features, *Pol* incorporates reverse transcriptase (RT), ribonuclease H (RH), and integrase (IN) activities. AluYa5 (~ 280 bp) is composed of left (L) and right (R) monomers divided by an adenosine-rich region and is followed by a polyadenine tail (A_n_). SVA_F_ (~2 kbp) is a composite element that brings together a variable number of CCCTCT hexamers, an *Alu*-like sequence, a variable number tandem repeat (VNTR) region and SINE-R, a sequence strongly resembling a portion of the HERV-K *Env* gene and 3′ LTR. L1HS (~6 kbp) is the only autonomously mobile human TE. It encodes two sense ORFs (ORF1 and ORF2) and an antisense ORF (ORF0) that may boost its mobility [29]. ORF2p possesses endonuclease (EN) and reverse transcriptase (RT) activities. Black arrows indicate known promoter elements. Elements are not depicted to scale. **b** HERV family statistics [30–32]. **c** HERV retrotransposition model, adapted from [33]. A provirus mRNA is transcribed and translated into a Gag-Pro-Pol fusion protein. Gag is cleaved by Pro to generate a virus-like particle, containing the fusion protein and ERV mRNA. A specific tRNA binds the primer binding site (PBS) to promote reverse transcription, producing a cDNA. The cDNA forms a complex with integrase (IN) to integrate into a new genomic site. **d** Internal homologous recombination events can occur between 5′ and 3′ LTRs, resulting in loss of the provirus internal region and one LTR, to generate a solo LTR. **e** Long- and short-read sequencing approaches differ in their ability to discriminate HERV proviral and solo LTR alleles

ERVs are retrotransposons, a type of transposable element (TE) that spreads throughout the genome via a copy-and-paste mechanism [28]. Three retrotransposon families (Fig. 1a) are mobile in humans: long interspersed element-1 (LINE-1, or L1), *Alu* (a type of short interspersed element, or SINE) and the composite element SINE-VNTR-*Alu* (SVA, where VNTR stands for variable number of tandem repeats) [28]. Human ERVs (HERVs) (Fig. 1a) appear presently incapable of retrotransposition, despite identification of polymorphic ERV insertions and HERV-K (HML-2) family copies with intact open reading frames (ORFs) [30, 31, 34–41]. Immobile ERVs, and particularly their flanking long terminal repeats (LTRs), can nonetheless contain promoter [21, 26, 27, 42–44] and enhancer elements [13, 16, 45], provide an extensive repertoire of transcription factor binding sites [46–48], and attract DNA and histone modifying complexes [49–54]. These features imbue ERVs with substantial capacity to regulate the expression of gene networks required for normal cell function. Indeed, the apparent frequency of ERV domestication is sufficient to support proposed models of predominant ERV cooperation, rather than escalating conflict, with the host genome [19, 22, 24, 33, 54, 55].

ERVs may elicit local and genome-wide transcriptional responses that result in immune activation in the context of disease, and ERV-derived regulatory elements govern aspects of the interferon response integral to innate immunity [16]. At present, the use of epigenetic therapies to induce ERV activation and downstream immune signalling in tumor cells, even if mechanistically unresolved, holds significant clinical potential [56, 57]. The motivation for this review is to dissect and disseminate recent evidence of ERV-associated oncogenesis, with emphasis on emerging therapies that target ERVs.

## A human ERV census

The reference genome assembly contains nearly 450,000 ERV-derived sequences stratified into nearly 100 families based on common features [1]. All ERV families discovered in humans were subsequently found in other primates, although some younger HERV loci are not conserved in other species [36, 38]. An intact HERV provirus is minimally composed of 5′ and 3′ LTRs flanking an internal *Gag*-*Pro*-*Pol* polyprotein-coding sequence [58] (Fig. 1a). Gag is cleaved by the protease (Pro) to generate viral particle structural proteins, whereas Pol encodes reverse transcriptase, integrase, and ribonuclease H enzymatic activities. Although HERVs can contain a remnant envelope (*Env*) gene, and other accessory genes, they are in general not infectious [59–61] and, due to germline ORF mutations, also lack potential for intracellular mobility. There is evidence for ongoing ERV retrotransposition in other mammals [62, 63]. In mouse, intracisternal A-type particle (IAP) ERVs are devoid of a functional *Env* gene and lack the capacity for virion formation and reinfection, but have become capable of retrotransposition via the formation of intracellular particles [63]. There is no evidence for such an adaptive process occurring in humans, with the possible exception of the HERV-K family. The reference genome contains at least 600 annotated HERV-K copies [30, 31] and population-scale analyses have found up to six polymorphic HERV-K loci per individual genome, including rare HERV-K proviruses with a full complement of intact *Gag*, *Pro*, *Pol*, and *Env* ORFs [9, 30, 31, 35–40, 60] (Fig. 1b). Reconstructed HERV-K lineage progenitor elements are infectious in vitro [60, 64, 65], and endogenous HERV-K mRNAs and proteins may be expressed in germline cells [9, 66, 67]. Genomic analyses of familial trios have not, however, recorded any robust de novo HERV-K insertions. HERV-K is therefore at most mobile under extremely limited settings, for instance due to reactivation of infectious HERV-K viral particles.

L1 facilitates all endogenous retrotransposition in human cells, including that of *Alu,* SVA, and other cellular RNAs [28] (Fig. 1a). L1 and HERVs were at some point likely both mobile in modern humans [35, 68] and each family may occasionally assist the other to retrotranspose [32, 69]. HERV LTR and L1 5′ UTR promoters can each drive transcription of adjacent genes [21, 70, 71]. However, L1 and HERVs differ in their retrotransposition strategies and potential *cis*-regulatory impact. As expected for a retroviral provirus, HERV mRNA transcription appears to mainly initiate toward the 3′ end of the 5′ LTR [72] and is followed by cytoplasmic reverse transcription (Fig. 1c). Whereas HERV insertions are depleted from gene-rich regions, particularly those in the same orientation as the host gene, reconstructed HERV-K proviruses exhibit moderate preference for intragenic integration [64, 65, 73]. By contrast, L1 mRNA transcription is mainly initiated from the first nucleotide of its 5′ UTR, and reverse transcription leading to L1 integration is primed from the nuclear genome [72, 74]. The L1 endonuclease has no apparent preference for gene-rich regions, and sense-orientated intragenic L1 insertions are more likely to be deleterious than their antisense counterparts [75]. HERV and L1 transcriptional regulation, as well as their retrotransposition-mediating protein complexes, are therefore distinct, even if in some situations they may be targeted by the same host defense pathways [76]. A further important consideration is that less than 2% of reference genome L1s retain a 5′ UTR, owing to the prevalence of L1 5′ truncation during integration. For these reasons, even if HERVs are presently immobile, they are arguably more likely than L1 to perturb gene regulation.

At least 85% of reference genome ERV instances are solitary (or “solo”) LTRs [1, 4, 31]. Solo LTRs arise from homologous recombination between ancestral 5′ and 3′ proviral LTRs, where the intervening protein-coding sequence is deleted [31, 41, 77] (Fig. 1d). Most ERV families are almost exclusively composed of solo LTRs and, amongst HERVs, these genome structural variants are still occurring. HERV-K and older HERV-H and HERV-W alleles present as a provirus in some individuals, and as a solo LTR in others, have been documented [31, 38, 39, 41]. These variants have the potential to perturb gene regulation, for instance by attracting repressive complexes [78] or, as exemplified by the pluripotency marker *ESRG*, remove exons overlapping a HERV internal sequence from a transcriptional unit [11, 31]. Given that solo LTRs and proviral LTRs can be difficult to distinguish with short-read sequencing [37] (Fig. 1e), the uptake of long-read technologies [79, 80] by cancer genomics and population resequencing consortia is likely to substantially expand the number of cataloged polymorphic solo LTRs. Although likely to be rare, these may include disease-associated variants.

## ERV wiring of developmental transcription networks

**Fig. 2:**
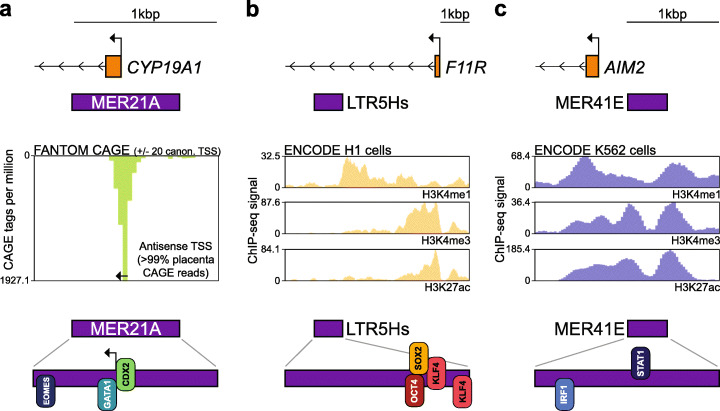
ERV regulatory element co-option. **a** An upstream MER21A LTR provides an alternative placenta-specific promoter to *CYP19A1* [15]. **b** A HERV-K solo LTR (LTR5Hs) enhancer located in the first intron of *F11R* [10]. **c** *AIM2* expression is enhanced by an adjacent MER41E LTR [16]. Note: each panel displays, from top to bottom, the first exon of a protein-coding gene, the position of an adjacent regulatory LTR, transcriptome (**a**) or histone modification (**b,c**) sequencing data, and a magnified view of relevant transcription factor binding sites in each LTR. Histone modification (H3K4me1, H3K4me3, and H3K27ac) profiles were obtained from ENCODE via the UCSC Genome Browser [2]. Transcriptome data in the form of cap analysis gene expression (CAGE) reads generated by the FANTOM consortium were visualized using the ZENBU genome browser [91]

The relevance of ERVs to pathogenesis is underscored by their contributions to normal development and human biology. HERV-derived regulatory elements are bound by transcription factors and activated during preimplantation development modelled in vitro [9, 20, 81, 82] (Table 1). Presumably, this aided the retroviral ancestors of HERVs in being expressed and endogenously amplified once they had accessed the germline [33]. HERVs display distinct, embryonic stage-restricted transcriptional profiles [83] and can harness transcription start sites to adjacent genes [21, 44]. For instance, HERV-L elements are upregulated during embryonic genome activation and can be bound by the transcription factor DUX4 to serve as alternative promoters for cleavage stage genes [84]. Dux, the functional murine orthologue of *DUX4*, binds the related mouse ERV family, MERV-L [84, 85]. MERV-L provides numerous alternative promoters for genes expressed at the 2-cell stage, when mouse embryonic transcription begins [86–88]. HERVs contribute exons to long noncoding RNAs, such as *ESRG*, *HPAT5*, and *linc-ROR*, which are upregulated by OCT4 and other pluripotency factors and may in turn act as molecular sponges for miRNAs limiting pluripotency factor expression [7, 8, 11, 89, 90]. The trophoblast cells of the placenta also express ERVs. For example, a primate MER21A LTR inserted upstream of the *CYP19A1* aromatase gene promotes transcriptional initiation of a highly abundant and placenta-specific *CYP19A1* mRNA (Fig. 2a) [15, 26]. *CYP19A1* generates ~ 0.3% of the capped mRNA found in human placental tissue, and yet is not expressed in the non-primate placenta [15, 91]. ERVs in sum provide an extensive catalog of alternative and canonical transcription start sites for genes expressed in early development, many of which are human-specific [21, 44].

Beyond serving as promoters during early development, HERV-H and HERV-K sequences appear to more frequently behave as enhancers (Table 1). The genome contains an estimated 800,000 elements bearing an enhancer biochemical signature [2], although only a fraction of these have been functionally validated as bona fide enhancers [92]. Enhancers may regulate both proximal and distal genes, and as enhancer-associated noncoding RNAs are widespread, the mechanisms by which enhancers influence gene expression are often not straightforward to resolve [92]. Candidate enhancer elements are disproportionately likely to overlap HERV-H sequences (Table 1). HERV-H copies are highly transcribed in the pluripotent cells of the blastocyst [7, 9, 11] and demarcate boundaries of open chromatin [12]. CRISPR-Cas9 mediated deletion or repression of HERV-H loci can alter the expression of upstream genes in the same topologically associated domain, in line with HERV-H copies often functioning as enhancers over longer genomic distances (Table 1) [10, 12]. HERVs supply transcription factor binding sites to the network of regulatory elements governing pluripotency [9, 10, 33, 93, 94]. By example, the consensus HERV-K LTR (LTR5Hs) harbors an OCT4 binding site, and in embryonic stem cell cultures, OCT4 directly binds and transactivates DNA hypomethylated LTR5Hs sequences [9]. HERV-K transcription, in contrast to HERV-H, begins in the embryonic genome at the 8-cell stage [9, 93]. However, as per HERV-H, HERV-K can provide proximal and distal enhancers (Table 1). One such example is an intronic HERV-K LTR enhancer of the pluripotency marker gene *F11R* [10] (Fig. 2b). Concomitant with the global acquisition of somatic heterochromatin, HERV transcription is downregulated post embryonic implantation [12, 93]. Although further work is needed to understand the developmental function and dispensability of HERV expression in vivo, it is clear that HERV regulatory elements are intimately and reciprocally linked with early embryonic development.

Despite their global postimplantation downregulation, some ERV families are predicted to contribute to lineage-specific enhancer networks [13, 25]. In particular, ERV cis-regulatory elements facilitate the interferon gamma (IFNG) response [16, 17, 20, 95] with the primate-specific MER41 family being the best characterized example [16, 96]. MER41 can harbor binding motifs for the transcription factors IRF1 (interferon regulatory factor 1) and STAT1 (signal transducer and activator of transcription 1) [16, 96]. Upon IFNG induction, some MER41 copies are bound by STAT1 and IRF1 and exhibit H3K27ac enrichment, a property of active enhancers [2, 16, 91, 96]. In a seminal 2016 study, Chuong et al. found a MER41 element immediately adjacent to *AIM2*, which encodes an interferon-stimulated protein that detects double-stranded DNA (dsDNA) upon viral or bacterial infection, and one that can elicit an inflammatory response [97]. Crucially, the MER41 sequence contained the only STAT1 binding motif within 50 kbp of *AIM2* (Fig. 2c). CRISPR-Cas9 deletion of this MER41 instance prevented *AIM2* expression following IFNG induction and diminished the downstream inflammatory response. As well, Chuong et al. observed attenuated expression of several other interferon response genes upon deletion of nearby MER41 copies, suggesting recurrent co-option of this ERV family as IFNG-inducible proximal enhancers [16]. MER41 has also been found to participate in long-range chromatin interactions with genes involved in the IFNG response, raising the possibility that MER41 copies can act as distal enhancers [98]. The contribution of MER41 to IFNG signalling elucidated by Chuong et al. provides clear precedent for ERVs shaping somatic transcriptional networks [95] and offers a framework by which ERV regulation could be probed and manipulated elsewhere.

## Pathways to modulate ERV activity

Host genomes maintain numerous systems to modulate ERV activity, as thoroughly reviewed elsewhere [51, 76, 99, 100]. Here we will briefly focus on the ERV control mechanisms that presently show the most potential as targets for genetic or pharmacological therapies (Table 2). Programmed mammalian ERV repression is enacted through the interconnected PIWI-interacting RNA (piRNA), DNA (CpG) methylation, chromatin packaging, and KAP1 (TRIM28) pathways, amongst others [76, 111]. piRNAs are predominantly found in male germ cells and function to silence TEs, in part, by recruiting DNA methylation [112, 113]. PIWI proteins are necessary for de novo DNA methylation of ERVs during male gametogenesis [112, 114, 115]. piRNAs are generally not encountered in mammalian somatic cells [113, 116]. In the embryo, KRAB-Zinc Finger Protein (KZFP) binding initiates silencing of ERVs and other TEs [52, 54, 117]. Some 350 KZFPs show highly orchestrated expression patterns that parallel ERV expression dynamics [22, 49, 52–54, 93, 118]. Specific KZFPs directly bind individual ERV copies and elicit silencing through recruitment of KAP1. KAP1 is a scaffold protein that engages effectors involved in heterochromatin formation [52, 119, 120]: the histone H3, lysine 9-specific methyltransferase SETDB1; the nucleosome remodelling and deacetylation (NuRD) complex; heterochromatin protein 1 (HP1); and DNA methyltransferases [121–124]. Notably, each of these effectors can serve as an enzymatic target for the therapeutic modulation of ERV activity. Following their deposition in the embryo, DNA methylation and H3K9me3 work in concert to ensure stable and permanent ERV repression [52, 119, 125].

**Table 2:** Therapeutic agents shown to stimulate ERV expression in preclinical models of cancer

Compound	Target	Cancer model	Possible ERV-related clinical effects	References
5-azacytidine 5-aza-2-deoxycytidine (5-aza-CdR)	DNMT1	Epithelial ovarian cancer cell lines B16-F10 mouse melanoma model Colorectal cancer cell line	dsRNA mediated viral mimicry Sensitizes cells to checkpoint inhibitor α-CTLA-4	[101, 102]
GSK-LSD1	LSD1 (KDM1A)	Breast cancer cell line B16 mouse melanoma model	dsRNA viral mimicry Sensitizes cells to checkpoint inhibitor α-PD-1	[103]
Vorinostat Pracinostat + 5-aza-CdR	pan-HDAC HDAC class I/II/IV HDAC + DNMT1	Lung cancer cell line	Activates ERV cryptic transcription start sites Stimulates interferon response	[42]
GSK126 GSK343	EZH2	Resistant chemorefractory small-cell lung cancer Taxane-resistant triple negative breast cancer cell lines	Promotes dsRNA production Alters innate immune signalling and promotes EMT Induces viral mimicry	[104, 105]
UNC0638 + 5-aza-CdR	G9a + DNMT1	Ovarian cancer cell lines	Induces viral mimicry	[106]
MC180295	CDK9	Colorectal cancer cell line Ovarian cancer cell line injected into mice	Induces viral mimicry Sensitizes cells to checkpoint inhibitor α-PD-1	[107]
Abemaciclib	CDK4/6	Mouse breast cancer models	Inhibits DNMT1 expression thereby inducing viral mimicry	[108]
Vitamin C	TET enzymes	AML, colorectal, breast, and liver cancer cell lines	Induces viral mimicry	[109]
RRX-001	Hemoglobin	Colon cancer cell lines	Decreases DNMT expression to induce viral mimicry	[110]

KAP1-mediated ERV silencing is robust yet not universal. Recent long-read methylome data generated from adult tissues suggest DNA methylation is lower at HERV-K elements than at other recently-mobile TEs or non-TE sequences [79]. HERV-K copies are unusually likely to present tissue-specific chromatin marks consistent with active regulation [126]. More generally, ERVs can promote tissue-specific transcription regulated by KZFPs in a wide range of normal tissues [15, 21, 22, 26, 53, 54, 70, 127, 128]. Many KZFP binding sites are also bound by tissue-specific transcription factors and display biochemical hallmarks of enhancers [54]. Furthermore, in T cells, KAP1 corepressor binding is differentially maintained across subsets of repressed ERVs [18]. These data collectively lend themselves to an extended model of KAP1 silencing in which lineage-specific KZFP expression facilitates tissue-specific ERV control and domestication [22, 53, 54]. KAP1-mediated repression is therefore an appealing target to precisely modulate ERV activity, especially as CRISPR-dCas9 fusion proteins have proven adept for this purpose in vitro [10, 12, 45, 92, 93].

## A complex interplay between ERVs and innate immunity

New viral infections, ERVs, and the innate immune system, coincide in vivo. Exogenous retroviruses can drive pathogenesis directly by insertional mutagenesis or introducing new regulatory elements, and indirectly by activating ERVs [43, 45, 129, 130]. A major cause of liver cirrhosis and hepatocellular carcinoma, for example, is hepatitis B virus (HBV) infection. In liver tumor genomes, HBV integrants can provide oncogenic enhancers and promoters, alongside an overarching epigenomic landscape that fails to repress TEs [43, 79, 131–133]. Infection leading to chronic inflammation and disease is in the liver, and in many other contexts, a well-established aetiological paradigm, with ERVs proposed to impact immune physiology and pathology [134]. ERV expression is also associated with inflammatory diseases of the central nervous system, including multiple sclerosis and amyotrophic lateral sclerosis, as discussed in detail elsewhere [135–137]. For the purpose of this review, we will focus on the convergence of innate immune signalling pathways and ERV expression products, which is relevant when considering ERV activity in tumors.

ERVs sit at the interface of self:non-self recognition. In what has been called viral mimicry, ERV expression can elicit host cell immune signalling via induction of viral defense pathways [101, 102, 134, 138]. One proposed explanation for this immune response is the recognition of ERV Env proteins and dsRNAs by pattern recognition receptors (PRRs) [139, 140]. Many exogenous viruses produce dsRNA at some stage of their replication cycle. Viral defense pathways are activated when PRRs recognize so-called pathogen-associated molecular patterns (PAMPs), such as dsRNA, and initiate immune signalling [141]. dsRNA is sensed by endosomal TLR3 and cytosolic MDA5-MAVS pathways, activation of which results in the induction of type I and type III interferon signalling and increased immunogenicity [142]. Induction of interferon signalling may initiate a positive feedback loop, in which ERVs can be bound and activated by immune effectors such as STAT1, IRF1, and NFκB, exacerbating ERV expression and the interferon response [16, 95, 96, 134, 143]. ERV protein products can also trigger proinflammatory cytokine signalling via TLR4, which is sustained by a positive feedback loop of HERV expression, further TLR4 activity, and chronic inflammation [139]. In mouse, ERV reactivation has been observed when immune signalling pathways are perturbed, upon deletion of nucleic acid sensors TLR7, TLR3, and TLR9 [134, 140] and in immunodeficient animals [138]. The interconnected nature of this regulation demonstrates a key point: because ERVs are so entwined in the innate immune system, their contributions to pathogenesis, as causal agents or bystanders upregulated by inflammation, are difficult to untangle.

## ERV-mediated oncogenesis

Tumorigenesis significantly resembles the corrupt reversion of cells to an earlier developmental state [144]. As for the preimplantation embryo, TE reactivation is seen in many cancers and can influence tumor genome stability [125, 145–150]. Extensive chromatin remodelling occurs upon malignant transformation. DNA methylation is redistributed across the genome and, in general, TEs are hypomethylated [79, 132, 145]. However, TEs do not all adhere uniformly to these trends. Recent analyses suggest a significant cohort of individual TE copies, including ERVs, are consistently unchanged, or more methylated, in cancer than in the corresponding normal cell type [79]. Perhaps these ERVs are influenced unexpectedly by another layer of epigenetic control [53, 109, 119, 125] or are already hypomethylated prior to tumorigenesis [79, 126, 132, 150, 151]. These observations have two major implications. Firstly, any cancer therapy intended to target ERV families must consider that each of its copies, including those proximal to key cancer genes, may not behave in the same way. Secondly, as epigenetic repression may be more, or less, enforced on each ERV locus, the accessible complement of ERV regulatory elements is changed, making both tumor suppressor gene downregulation and oncogene upregulation possible at a time and place that alters the course of disease [43, 45, 127, 143, 144, 152, 153].

**Fig. 3:**
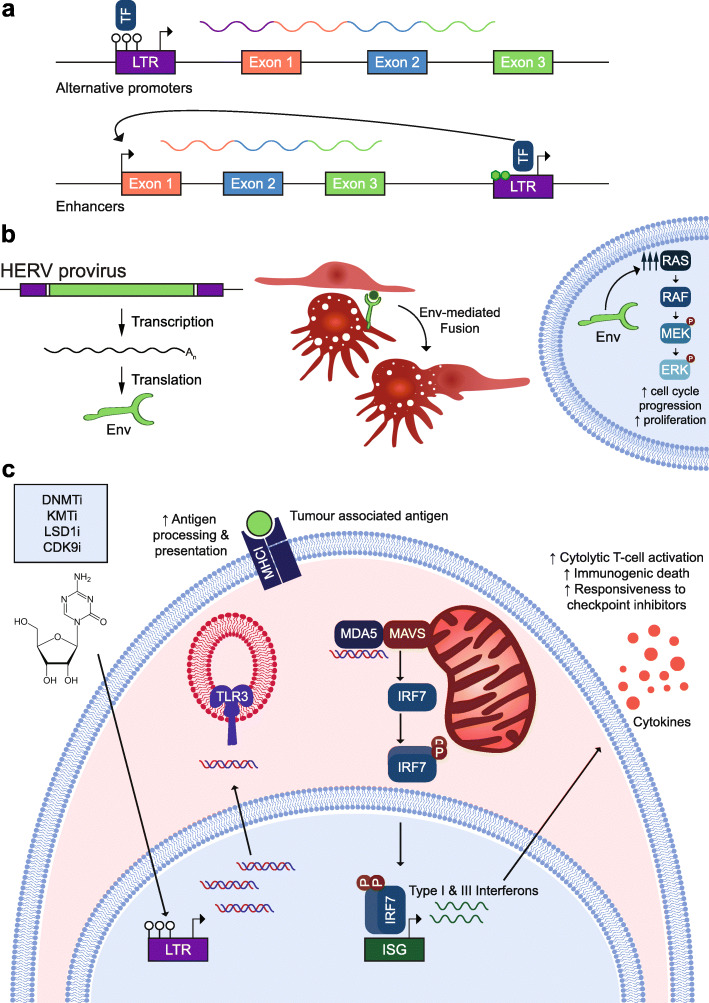
ERVs contribute to oncogenesis and may be targeted therapeutically. **a** Onco-exapted ERVs can regulate host gene expression in tumors. Hypomethylated ERV LTRs can be bound by transcription factors (TF) and serve as alternative promoters to induce the expression of oncogenes contributing to oncogenesis (top). They may also be decorated with H3K27ac and H3K4me1 (light green) and act as enhancers to drive the expression of adjacent or distal genes (bottom). Note: empty white circles represent unmethylated CpG dinucleotides. **b** HERV proteins, such as Env, can be expressed from intact HERV provirus ORFs in cancer cells (left). In breast and endometrial carcinoma models, Env has been detected on the cell membrane and is able to mediate cellular fusion with endothelial cells [154, 155] (middle). Env can promote tumorigenesis, for example by stimulating the RAS/RAF/MEK/ERK pathway in breast cancer models [156]. **c** Proposed model for therapeutic targeting of ERV-induced viral mimicry to promote immune detection of tumor cells. ERV reactivation can be induced by a number of therapeutic agents that target chromatin modifying enzymes (blue box). Derepression of ERV LTRs results in the production of dsRNA molecules, which can be detected by cytosolic dsRNA sensors TLR3 and MDA5 [101, 102, 134]. MDA5 binds to MAVS in the mitochondria and stimulates a signalling cascade which promotes the phosphorylation, dimerisation, and nuclear translocation of interferon regulatory factors (IRFs). IRFs drive a type I/III interferon response to spur cytokine production and increase the immunogenicity of the cell. Viral mimicry synergises with production and presentation of ERV-derived tumor associated antigens (green dot) via MHC-I, to increase the visibility of tumor cells for immunogenic death by cytolytic T cell activation

In tumors, reactivated TEs can promote oncogene expression, contributing to disease progression in a phenomenon termed onco-exaptation [152]. Again, much like in development, ERVs can serve as alternative promoters for nearby genes in malignant cells (Fig. 3a) [127, 152, 157, 158] and cryptic transcription start sites within ERVs can be employed to produce aberrant protein-coding mRNAs, including those with novel ORFs [42, 157]. Analyses of many cancer types suggest TE onco-exaptation is relatively common [43, 45, 127, 133, 152, 159]. This is well demonstrated by an upstream MaLR (THE1B) LTR sequence providing an alternative promoter to *CSF1R* in Hodgkin’s lymphoma, a B cell cancer [143]. In healthy myeloid cells, *CSF1R* is under the control of its canonical promoter and an intronic enhancer bound by the transcription factor PU.1. In Hodgkin’s lymphoma cells, *CSF1R* is upregulated, despite absence of PU.1 [143]. As resolved by Lamprecht et al., loss of DNA methylation and downregulation of the corepressor CBFA2T3 alleviates THE1B repression, allowing it to initiate transcription of an aberrant *CSF1R* mRNA [143]. Furthermore, Lamprecht et al. found the THE1B promoter was activated by NFκB binding, highlighting the synergy between chromatin pathways and ERV transactivators.

Alongside alternative promoter function, ERVs may also be onco-exapted as enhancers, as recently shown by Deniz et al. in an elegant study of acute myeloid leukemia (AML) [45]. Here, an initial epigenomic survey in AML samples returned a number of differentially accessible ERV families, including HERV-K, marked with H3K27ac and H3K4me1 and bound by AML-associated transcription factors, raising the possibility of enhancer function (Fig. 3a) [45]. CRISPR-Cas9 deletion of several ERVs in vitro downregulated adjacent AML-expressed genes. Importantly, targeted CRISPR-dCas9 mediated silencing of LTR2B, the candidate ERV (HERV-E) family exhibiting the greatest AML-restricted accessibility, led to impaired cell growth in AML cell lines. Further, deletion of an LTR2 (a close relative of LTR2B) copy annotated as an *APOC1* promoter [160], but matching an enhancer profile in immune cells [45], reduced cell proliferation and increased apoptosis. Together, these data from Deniz et al., reinforced by robust CRISPR-Cas9 based experiments, clearly depict ERVs as enhancers for genes impacting AML progression and patient survival [45]. Their results also suggest, as per normal development, that ERVs are generally more influential in AML as enhancers than as promoters, a pattern that may hold across most cancer types [43].

HERV-K Env, Rec, and Np9 proteins, as well as HERV-W Env, are detected in tumors and are proposed to have oncogenic properties [136]. Their hypothesized tumorigenicity is via routes similar to how they are co-opted in development. Akin to HERV-W Env-derived Syncytin mediation of cell fusion in the placenta, HERV-W protein upregulation in breast and endometrial cancers has been associated with increased cell-cell fusion, occurring with cancer progression, metastasis, and chemoresistance (Fig. 3b) [5, 154, 155]. There is also evidence that Env, as well as the HERV-K proviral accessory proteins, Rec and Np9, stimulate or interfere with endogenous cell signalling pathways critical for cellular growth and proliferation (Fig. 3b) [156, 161–163]. While HERV proteins are considered insufficient to induce transformation, their contribution to oncogenesis underlines their therapeutic potential [161]. Targeting HERV-K Env proteins with antibodies appears to inhibit cell growth and increase apoptosis in breast cancer cell lines, and reduce growth of xenograft tumors in mice [164, 165]. Intriguingly, regression of metastatic renal cell carcinoma was reported following allogeneic stem cell transplantation, albeit for a single patient, in which donor T cells recognized an HERV-E encoded antigen [166]. Thus, while ERV-mediated gene dysregulation offers a clearer path to onco-exaptation, the products of residual ERV protein-coding potential may be a more immediate and tractable therapeutic target.

## Therapeutic modulation of ERV expression in cancer

Cancer therapies intended to activate ERVs have been intensely explored in recent years [56]. Despite showing promise, it remains unclear as to whether inducible ERV activity can be leveraged to treat solid tumors, especially provided potential for ERV onco-exaptation and other unintended consequences [45, 127, 143, 152]. The prevailing mechanistic model here, based on viral mimicry, is that TE activation can provoke an anti-tumor immune response, as described in cancer cells treated with DNA methyltransferase inhibitors (DNMTis) [101, 102, 167]. DNMTis are used as a broad-brush approach to modulate the epigenome in malignant cells and are thought to reactivate inappropriately silenced tumor suppressor genes [57]. Along with other epigenetic therapies, DNMTis have proven effective against hematological malignancies [57]. Two 2015 studies reported that DNMTi treatment stimulates ERV dsRNA production in solid tumor models, triggering an immune signalling cascade mimicking that of an exogenous virus (Fig. 3c) [101, 102]. This viral mimicry has since been achieved by inhibiting histone deacetylation complexes, lysine demethylases, and histone methyltransferases [103, 168, 169] (Table 2) in a range of solid tumor models, including melanomas, and colorectal and ovarian cancers [101–103].

One appealing aspect of the ERV viral mimicry model is its integration with more established immune vulnerabilities exploited to treat hematological cancers [57]. In this system, ERV dsRNAs are recognized as non-self, triggering type I and type III interferon signalling. Interferon signalling can promote anti-tumor immunity through the extrinsic stimulation of cytotoxic lymphocyte populations in the tumor microenvironment, and via tumor cell intrinsic effects, including growth inhibition, modulation of apoptosis, and induction of further mediators of immune signalling [170]. While in principle the innate immune pathways stimulated by HERV products are those that are employed upon exogenous retroviral infection, further work is needed to elucidate the specific immune response to ERV induction in tumor cells. Viral mimicry in tumors has largely been inferred from type I/III interferon gene expression signatures. Consistent with an interferon response, several studies have observed infiltration of cytotoxic and helper T cells in the tumor microenvironment upon ERV induction in mouse models of cancer [103, 104]. Treatment with DNMTis to induce viral mimicry enhances CD8^+^ T cell activation and detection of cytotoxic mediators in vitro [170, 171]. ERV expression may also contribute to an adaptive immune response through the production of tumor associated neoantigens (Fig. 3c) [172]. HERVs can produce epitopes presented as MHC-I-bound peptides at the surface of tumor cells, which can increase tumor cell visibility to immune surveillance, resulting in a cytolytic T cell response [166, 173]. Crucially, enhanced immunogenicity can overcome resistance to checkpoint blockade inhibitors [57] in the treatment of solid tumors [174]. For example, CTLA-4 and PD-1 are immune checkpoint proteins that negatively regulate cytotoxic T cell response [175]. These molecules are upregulated in tumor cells that evade immune targeting [56]. Monoclonal antibodies against CTLA-4 and PD-1 have successfully inhibited immune checkpoint response in the clinic, enabling immune clearing of tumor cells [176, 177]. However, patient response to immune checkpoint inhibitors varies widely [178]. Promising results from melanoma mouse models suggest ERV viral mimicry brought about through inhibition of DNMT1 or the histone demethylase LSD1 can potentiate the anti-tumor effects of CTLA-1 and PD-1 inhibitors, respectively [101–103]. ERV-induced viral mimicry could thus prove useful in combination therapies designed to overcome resistance to immune checkpoint inhibitors, for which clinical trials are ongoing [56, 57, 179].

ERV demethylation and transcription as a trigger for innate immune activation is proposed to explain the clinical impact of DNMTi treatment [56, 101, 102]. While an exciting and worthwhile concept, we consider this position with some caution, and doubly so considering the clinical implications. Moving the focus away from ERVs, multiple studies have tied viral mimicry to LINEs and SINEs [167]. In colorectal cancer cells, viral mimicry has been primarily associated with inverted *Alu* copy dsRNAs [180], whereas in a DNMTi-treated glioblastoma cell line, SVA and ERV upregulation are comparable [181]. In hematological datasets, expression from a diverse array of TEs, including ERVs, SINEs, and LINEs, has been linked with viral mimicry upon DNMTi treatment [182]. Inactivation of the HUSH complex component MPP8 leads to both ERV and L1 upregulation, with the latter associated with dsRNA production and a type I interferon response [183]. Global ERV expression levels have been found to not predict clinical response of myelodysplasias treated with DNMTis [184]. Taken together, these results suggest that the ERV-centric model of viral mimicry upon treatment with DNMTis may be too simplistic. There is a pressing need, in our view, to move beyond inference of viral mimicry from correlations with TE family transcript levels. One approach could involve ectopic expression of various TEs upregulated in DNMTi-responsive patients, as shown to yield a gene expression signature consistent with innate immune activation [182, 183]. Arguably the most direct evidence for TE-induced viral mimicry to date comes from an RNA protection assay for MDA5 in DNMTi-treated colorectal cancer cells [180]. Here, *Alu*, and not ERV or L1, transcripts were significantly enriched in MDA5-protected RNA, which argues that *Alu*s are the main source of immunogenic RNA in this model [180]. Such experiments expand the viral mimicry model to include non-ERV TEs as potential therapeutic targets in cancer [167, 180, 183]. Further experiments are required to resolve the individual TE copies that are capable of inducing viral mimicry, and directly test their immunogenicity.

Advances in genome and epigenome editing, as well as sequencing technologies, provide the means to reach this level of resolution. The epigenomic analyses presented to date have tended toward a summary view of TE families. High-resolution DNA methylation and histone modification profiles for specific ERV, L1, and *Alu* loci with and without exposure to DNMTis would be informative, particularly if integrated with long-read transcriptomic analyses of dsRNAs and their precursor molecules. As well, long-read methylome data indicate HERV CpG methylation is already lower than that of other TEs and the remaining genome in normal tissues, and may be less reduced in tumor cells [79]. It is therefore possible that in many tumor cells DNA methylation may not be the predominant mechanism employed to limit ERV transcription [125]. Histone deacetylase inhibitors and lysine methyltransferase inhibitors strongly synergise with DNMTis to activate ERVs [42, 106], while repressive chromatin pathways can have compensatory effects [105, 119, 125, 185, 186]. These considerations are likely to impact the success of ERV-focused cancer therapeutics.

Given recent evidence implicating a number of TE families in triggering viral mimicry, it would now be beneficial to directly dissect and modulate ERV activity in preclinical tumor models. A challenge in targeting ERVs, and TEs in general, is specificity; most studies of ERV function in cancer have examined entire ERV families, whereas few have targeted specific loci [45, 101–103]. Data obtained from cancer cell lines have shown that in vitro-transcribed ERVs can trigger gene expression changes that indicate viral mimicry, and that different ERVs evoke different gene expression signatures [182]. This highlights the need to dissect ERV families and individual ERV loci in mechanistic explanations founded on viral mimicry. To be more clinically relevant, these studies could be expanded to study the modulation of ERVs and other endogenous TEs in situ via CRISPR-Cas9 and CRISPR-dCas9 strategies [45]. As CRISPR-Cas9 genome editing relies upon guide RNA (gRNA) identity to specific target sequences, and ERVs form families of closely related sequences, it is possible to CRISPR-Cas9 edit entire ERV families, as well as individual ERV loci [10, 12, 16, 45, 92]. In addition to providing a deeper and more precise understanding of ERV loci that could be immunogenic, this approach could indicate more examples involved in onco-exaptation, such as the *APOC1* LTR2 enhancer highlighted by Deniz et al. [45]. CRISPR-dCas9 fusion proteins can also be employed to bring activating (CRISPRa) and interfering repressor (CRISPRi) complexes to specific TE loci. This approach has proven particularly useful for ERVs, where activator (e.g., CRISPR-dCas9-VPR) and repressor (e.g., CRISPR-dCas9-KRAB) fusion proteins can be used to assess the downstream effects of modulating ERV activity [10, 12, 45, 92, 93]. In the context of cancer, the downstream consequences for dsRNA production and interferon signalling upon ERV activation could then be assessed. Even if various obstacles to their application to cancer still need to be addressed, the use of CRISPR-Cas9 tools will greatly facilitate hypothesis testing surrounding ERV regulation and function, particularly in instances inferred from epigenome data and correlated transcription [2, 20, 21, 43, 91].

Preclinical modulation of ERV expression has been pursued with agents that induce global changes to the cancer epigenome landscape (Table 2). Although many of these therapies are FDA approved for hematological malignancies, their utility against solid tumors is less established. Their lack of precision and selectivity are potential drawbacks, as epigenetic modifiers are ubiquitously expressed and of multifaceted function. Epigenetic therapies thus present a challenge to elucidate the mechanism(s) linking a given inhibitor with altered tumor cell phenotype. With this complexity in mind, it is perhaps unsurprising that the main upstream effectors of the viral mimicry pathway remain to be resolved with certainty [180]. The clinical success of epigenetic therapies rests on the greater dependence of tumor cells on the targeted chromatin modifiers to drive aberrant transcriptional programs, compared to homeostatic transcription in normal cells [57]. Specific ERV reactivation leading to an immune response is arguably a more exact objective, and one perhaps more easily reached via another strategy. For example, *KAP1* deletion has a more pronounced effect on the expression of ERVs than on protein-coding genes [18]. In mouse models of melanoma, *KAP1* knockout enhances ERV-mediated viral mimicry [187]. Developmental ERV expression occurs in windows of epigenetic reprogramming and is facilitated by transactivator binding [7, 9, 16, 46, 47, 81–83, 93]. Identification of ERV transactivators that are expressed in cancer, but not normal cells, could provide a therapeutic avenue to stimulate ERV expression with sufficient specificity to exclude other genomic elements. One caveat of this approach is that modulation of developmental ERV transactivators alone may not be immunogenic, as apparent for DUX4 [188]. Other barriers to the use of more precise epigenetic therapies against ERVs, perhaps involving CRISPR-dCas9 fusion proteins, are delivery and safety, which have been overcome for many FDA-approved DNMTis. A higher-resolution view of the chromatin and cell signalling pathways that converge to activate ERV expression in development and disease will likely illuminate new therapeutic targets.

## Future directions

Cancer, inflammation, and embryogenesis all bear witness to the impact of ERVs on human biology. Here we have attempted to bring forward exemplary studies that resolve and explain how ERV-derived nucleic acid and protein function in these contexts. The bulk of such phenomena are partially described, or fully remain to be uncovered. It is apparent that ERVs are expressed in a range of malignancies, including in response to therapeutic agents, and may serve as prognostic biomarkers [156, 189]. While the functions or mechanisms by which ERVs can contribute to pathology are frequently not well understood, new strategies focused on ERVs to treat common and deadly diseases hold obvious merit. To our knowledge, ERV modulation in preclinical studies of cancer, as well as of autoimmune and neurodegenerative conditions, has been approached globally, such as with epigenetic therapies or reverse transcriptase inhibitors. More precise, and higher-resolution, approaches illuminated by a deeper understanding of ERV regulation in somatic cells could accelerate development of ERV-focused therapeutics. Fortunately, long-read sequencing and CRISPR-Cas9 technologies have emerged as powerful tools to probe the causal contributions made by ERVs to pathology. While the role of ERVs, and other TEs [149, 180], in generating viral mimicry and inducible interferon responses in cancer are clearly an area of priority, similar experimental techniques could be applied to autoimmune and neurodegenerative diseases where ERV dysregulation is encountered. A comprehensive view of ERV regulation and function in normal cells, combined with the specific contributions made by ERVs to disease, is required to fully realize their exciting if still preliminary clinical potential.

## Supplementary Information

**Additional file 1.** Review history.
