# Supplementary material for: Endogenous retroviruses in the origins and treatment of cancer
Source: Genome Biol. 2021 May 10;22:147. doi: 10.1186/s13059-021-02357-4 (PMC8108463; doi:10.1186/s13059-021-02357-4)
Supplement: Supplementary file 1 — Additional file 1. Review history. [file 13059_2021_2357_MOESM1_ESM.docx]

Review History

**First round of review**

**Reviewer 1**

**Comments to author:**

Genome Biology

Review article: Jansz and Faulkner

This review is timely, well written and engaging to read with abundant references from the field. I have several suggestions to make it more complete and accessible to a broad readership.

1.In the introduction, it is stated that 'most ERVs entered the germline via retroviral infection of a distant ancestor…' It would be good to give the reader an idea of when the peak of ERV invasions occurred (PMID: 10729147 for example).

2.Page 2: 'Human ERVs (HERVs) appear presently incapable of retrotransposition…' It could be worth mentioning that mouse ERVs (IAP elements) have adapted to become capable of retrotransposition (rather than relying on re-infections of the germ-line), for example through loss of their envelope gene and re-targeting of capsids but there is no evidence of this happening in humans.

3.Page 2: 'For instance, remnant ERV proteins are more abundant in tissues obtained from multiple sclerosis patients than in controls, and may trigger cytokine feedback loops driving inflammation..' It would be good to clarify what is being referred to here: ERV nucleic acids may drive interferon responses and ERV proteins may be presented as antigens and drive T cells responses. Or ERV proteins may induce cell death and the production of damage-associated molecular patterns (DAMPs), which drive inflammation or is another mechanism referred to here?

4.Page 7: 'ERV co-option to provide species- and lineage-specific regulation of interferon gamma (IFNG) cytokine signalling'. I think this sentence would be clearer by removing the word 'cytokine' as it implies other cytokines, whereas the authors are just referring to interferon gamma and have already specified this.

5.Page 12: section on 'Therapeutic modulation of ERV expression in cancer': As well as DNMT1/LSD1 inhibition, inactivation of the HUSH complex component, MPP8 leads to accumulation of dsRNA and an IFN-beta response (PMID: 33144593). MPP8 regulates L1 elements, exhibits diverse mRNA expression in cancers compared to controls, and cancer samples from the Cancer Genome Atlas with downregulated MPP8 have an IFN dominant signature. This suggests that the HUSH complex may be a promising drug target for cancer to induce viral mimicry.

6. I found table 1 informative. It could be more accessible though if the most striking findings were colour-coded, for example, LTR7s serving as distal enhancers could be highlighted in one colour and the main CTCF-bound ERVs in another colour.

7. Overall, some of the text could be cut down to make the review a little more focused on cancer. Parts of the 'paths to ERV repression' section were a little unclear and could do with some changes for readability.

**Reviewer 2**

**Comments to author:**

Jansz and Faulkner review ERV co-option in development and innate immunity, the aberrant contribution of ERVs to tumorigenesis, and the wider biomedical potential of therapies directed at ERVs. The idea of ERVs involving in tumorigenesis goes back more than 20 years. In recent years, advancement in genomics allowed some of the earlier ideas to be reexamined, and the field is undergoing a rapid expansion. Thus, this review is timely. The review is overall well written. The authors gave a good introduction to ERVs in the human genome, followed by sections on what is known about ERVs in development, innate immunity, autoimmune diseases, and cancer. The key strength of this review is that it is fairly broad, covers many topics and provides an overview of the field.

Since the review focuses on cancer, in its current form it reads somewhat diffused and lack of depth. The discussion regarding cancer origin and treatment makes up less than half of the review (two cancer sections start on page 10). Although the earlier sections are well written, they are only loosely connected to the central thesis rather than being integrated, reducing the overall impact of the key message. The two sections on cancer as well as the future direction section could be more streamlined, go into more depth, and more importantly, should be much better synthesized in addition to surveying the field. I will use a few examples to illustrate my points.

In the "ERV wiring of innate immunity" section, the two paragraphs are disconnected. The first paragraph stops short at mentioning ERV impact immune physiology and pathology, whereas the second paragraph entirely focuses on the seminal work from Chuong 2016 on TE exaptation as enhancers responding to IFNG signaling through Stat1. I found it difficult to understand the key point from this section.

In the "ERV induced autoimmunity" section, it is not clear to me exactly how "useful crisis" is defined. Is it host defense? Is it adaptation? Evidences reviewed in this section do not always link to autoimmunity. In the first paragraph, the primary literature referenced to support ERV dsRNA -> viral defense pathway were based on scenarios that do not involve autoimmunity. In the second paragraph, it seems that HERV-W might trigger autoimmunity through TLR4. This pathway is only briefly mentioned and should be elaborated. Throughout this section, it's not clear whether/how ERV dsRNA contributes to autoimmunity. The authors should also comment on how the adaptive immune system might play a role. Are there endogenous antibodies/T cells specific to HERV-W? Will it contribute to pathogenesis?

In the "ERV mediated oncogenesis" section, the key statement "The deregulation of host genes by TEs has been termed onco-exaptation" is incorrect. It is not any deregulation. The essence of onco-exaptation is related to cancer evolution, as defined in the cited study, and this concept seems to have been left out of the entire review.

In the "therapeutic modulation of ERV in cancer" section, the immune perspective is over-simplified, and needs to be expanded and clarified. Line 34 - 43 of page 12 attempted to explain the immunology behind ERV dsRNA enhancing checkpoint blockade. However, it's unclear how this process happens. How does IFN signaling induce T cell activation? What is immunogenic cell death referring to? Does it refer to CD8 T cell mediated killing or necrosis/pyroptosis? What are the processes that happen inside the cancer cell? What are the processes in antigen presenting cells (APC)? In Fig. 3, How does Type I/III interferons increase antigen presentation? The 2 arrows in the figure from "Type I & III interferons" to "antigen processing & presentation" made it seem like an intracellular pathway. However, it's more likely to involve autocrine and paracrine pathways. Type 1/3 interferons production -> release -> bind to IFN receptor on cell surface -> induce interferon stimulated genes (ISGs) -> ISGs increase antigen processing and presentation.

One of the highlights of this review is to point out whether it is ERVs or Alus that triggers viral mimicry upon epigenetic therapy. This is an important point, and authors should be more comprehensive in reviewing literature (e.g. Ohtani et al., Cancer Research 2020, Activation of a Subset of Evolutionarily Young Transposable Elements and Innate Immunity Are Linked to Clinical Responses to 5-Azacytidine) and in proposing strategies so that the mechanism can be elucidated.

One of the biggest challenges in studying and targeting ERVs is related to the specificity. Numerous studies examined ERVs at family or subfamily level, whereas a few were able to target specific loci. This point should be made clearer with examples. Importantly, this is a future direction worth expanding. With emerging technologies, including long-read sequencing and genome / epigenome editing, more specifically, what kinds of future investigations can be possible?

Fig 2 needs y-axis scale for genome browser views if the real data were used.

Reviewer #1: Genome Biology

Review article: Jansz and Faulkner

*This review is timely, well written and engaging to read with abundant references from the field. I have several suggestions to make it more complete and accessible to a broad readership.*

*1.In the introduction, it is stated that 'most ERVs entered the germline via retroviral infection of a distant ancestor...' It would be good to give the reader an idea of when the peak of ERV invasions occurred (PMID: 10729147 for example).*

We have added the approximate time since peak colonisation and the suggested citation to the text on page2, line 10: “over 30 million years ago [2]”.

*2.Page 2: 'Human ERVs (HERVs) appear presently incapable of retrotransposition...' It could be worth mentioning that mouse ERVs (IAP elements) have adapted to become capable of retrotransposition (rather than relying on re-infections of the germ-line), for example through loss of their envelope gene and re-targeting of capsids but there is no evidence of this happening in humans.*

We have added a sentence on active retrotransposition in other mammals, and the suggested example in mice, on page 3, line 26: “There is evidence for ongoing ERV retrotransposition in other mammals [59, 60]. In mouse, intracisternal A-type particle (IAP) ERVs are devoid of a functional *Env* gene and lack the capacity for virion formation and reinfection, but have become capable of retrotransposition via the formation of intracellular particles [60]. There is no evidence for such an adaptive process occurring in humans, with the possible exception of the HERV-K family.”

*3.Page 2: 'For instance, remnant ERV proteins are more abundant in tissues obtained from multiple sclerosis patients than in controls, and may trigger cytokine feedback loops driving inflammation..' It would be good to clarify what is being referred to here: ERV nucleic acids may drive interferon responses and ERV proteins may be presented as antigens and drive T cells responses. Or ERV proteins may induce cell death and the production of damage-associated molecular patterns (DAMPs), which drive inflammation or is another mechanism referred to here?*

We agreed that this needed further clarification. However, when streamlining the manuscript to better focus on cancer in response to a comment from Reviewer #2, we have removed the above text describing ERVs in MS.

*4.Page 7: 'ERV co-option to provide species- and lineage-specific regulation of interferon gamma (IFNG) cytokine signalling'. I think this sentence would be clearer by removing the word 'cytokine' as it implies other cytokines, whereas the authors are just referring to interferon gamma and have already specified this.*

We agreed with this point, though we also removed this sentence when streamlining the text in response to a comment from Reviewer #2.

*5.Page 12: section on 'Therapeutic modulation of ERV expression in cancer': As well as DNMT1/LSD1 inhibition, inactivation of the HUSH complex component, MPP8 leads to accumulation of dsRNA and an IFN-beta response (PMID: 33144593). MPP8 regulates L1 elements, exhibits diverse mRNA expression in cancers compared to controls, and cancer samples from the Cancer Genome Atlas with downregulated MPP8 have an IFN dominant signature. This suggests that the HUSH complex may be a promising drug target for cancer to induce viral mimicry.*

We thank the reviewer for highlighting this study. We have incorporated it, along with several others, in our expanded discussion on ‘Therapeutic modulation of ERV expression in cancer’ starting at page 12, line 54: “Inactivation of the HUSH complex component MPP8 leads to both ERV and L1 upregulation, with the latter associated with dsRNA production and a type I interferon response [177].”

6. *I found table 1 informative. It could be more accessible though if the most striking findings were colour-coded, for example, LTR7s serving as distal enhancers could be highlighted in one colour and the main CTCF-bound ERVs in another colour.*

We included this table to illustrate that selected HERVs are more frequently marked as enhancers than as promoters or as insulators. We have now red/blue colour coded the more striking findings, which we hope better illustrates this point.

7. *Overall, some of the text could be cut down to make the review a little more focused on cancer. Parts of the 'paths to ERV repression' section were a little unclear and could do with some changes for readability.*

We agreed with these broader comments. Hence, we removed the section on ERVs in immune disorders of the nervous system, so that the review is more focused on cancer. We have edited the ‘Pathways to modulate ERV activity’ section (page 7, line 5) for readability, mainly by simplifying the text and modifying very long sentences.

*Reviewer #2: Jansz and Faulkner review ERV co-option in development and innate immunity, the aberrant contribution of ERVs to tumorigenesis, and the wider biomedical potential of therapies directed at ERVs. The idea of ERVs involving in tumorigenesis goes back more than*

*20 years. In recent years, advancement in genomics allowed some of the earlier ideas to be reexamined, and the field is undergoing a rapid expansion. Thus, this review is timely. The review is overall well written. The authors gave a good introduction to ERVs in the human genome, followed by sections on what is known about ERVs in development, innate immunity, autoimmune diseases, and cancer. The key strength of this review is that it is fairly broad, covers many topics and provides an overview of the field.*

*Since the review focuses on cancer, in its current form it reads somewhat diffused and lack of depth. The discussion regarding cancer origin and treatment makes up less than half of the review (two cancer sections start on page 10). Although the earlier sections are well written, they are only loosely connected to the central thesis rather than being integrated, reducing the overall impact of the key message. The two sections on cancer as well as the future direction section could be more streamlined, go into more depth, and more importantly, should be much better synthesized in addition to surveying the field. I will use a few examples to illustrate my points.*

These were excellent points about the overall structure of the review. In order to make the text more streamlined, we have heavily edited sections to better relate them to our central messages on ERVs and cancer. We have also decided to entirely remove the section on autoimmune disorders.

*In the "ERV wiring of innate immunity" section, the two paragraphs are disconnected. The first paragraph stops short at mentioning ERV impact immune physiology and pathology, whereas the second paragraph entirely focuses on the seminal work from Chuong 2016 on TE exaptation as enhancers responding to IFNG signaling through Stat1. I found it difficult to understand the key point from this section.*

To make this section less disconnected, we have moved mention of Chuong et al.’s work to where we discuss TE exaptation in development (page 6, line 36). We think this is a better fit, as our key point in discussing this work is that ERVs can contribute to regulatory transcriptional networks. This has allowed us to focus the ‘ERVs and innate immunity’ section on how ERV transcripts and encoded proteins interact with innate immune signalling pathways, which is essential to understand why ERVs are being targeted by cancer therapeutics.

*In the "ERV induced autoimmunity" section, it is not clear to me exactly how "useful crisis" is defined. Is it host defense? Is it adaptation? Evidences reviewed in this section do not always link to autoimmunity. In the first paragraph, the primary literature referenced to support ERV dsRNA -> viral defense pathway were based on scenarios that do not involve autoimmunity. In the second paragraph, it seems that HERV-W might trigger autoimmunity through TLR4. This pathway is only briefly mentioned and should be elaborated. Throughout this section, it's not clear whether/how ERV dsRNA contributes to autoimmunity. The authors should also comment on how the adaptive immune system might play a role. Are there endogenous antibodies/T cells specific to HERV-W? Will it contribute to pathogenesis?*

**We thought about this at length, along with Reviewer #2’s comments around the overall content** of the review, and removed this section entirely to make space to discuss cancer in more detail.

***In the "ERV mediated oncogenesis" section, the key statement "The deregulation of host genes by TEs has been termed onco-exaptation" is incorrect. It is not any deregulation. The essence of onco-exaptation is related to cancer evolution, as defined in the cited study, and this concept seems to have been left out of the entire review.***

We have rewritten this sentence to make sure we properly convey the concept of onco-**exaptation (page 9, line 45): “**In tumours, reactivated TEs can promote oncogene expression, contributing to disease progression in a phenomenon termed onco-exaptation [[144]](https://paperpile.com/c/Lk39Kd/kcWdI)**.”**

***In the "therapeutic modulation of ERV in cancer" section, the immune perspective is over­simplified, and needs to be expanded and clarified. Line 34 - 43 of page 12 attempted to explain the immunology behind ERV dsRNA enhancing checkpoint blockade. However, it's unclear how this process happens. How does IFN signaling induce T cell activation? What is immunogenic cell death referring to? Does it refer to CD8 T cell mediated killing or necrosis/pyroptosis? What are the processes that happen inside the cancer cell? What are the processes in antigen presenting cells (APC)?***

We have expanded and re-written this paragraph, starting at page 11, line 16. To our knowledge, these questions have not been addressed specifically with regards to ERVs in tumours. We have highlighted what little is known about the immune response to ERVs, and now included examples. We have also added a sentence on the general intrinsic and extrinsic **effects of IFN signalling. The relevant part of this paragraph now reads: “One appealing aspect** of the ERV viral mimicry model is its integration with more established immune vulnerabilities exploited to treat haematological cancers [54]. In this system, ERV dsRNAs are recognised as non-self, triggering type I and type III interferon signalling. Interferon signalling can promote anti-tumour immunity through the extrinsic stimulation of cytotoxic lymphocyte populations in the tumour microenvironment, and via tumour cell intrinsic effects, including growth inhibition, modulation of apoptosis, and induction of further mediators of immune signalling [163]. While in principle the innate immune pathways stimulated by HERV products are those that are employed upon exogenous retroviral infection, further work is needed to elucidate the specific immune response to ERV induction in tumour cells. Viral mimicry in tumours has largely been inferred from type I/III interferon gene expression signatures. Consistent with an interferon response, several studies have observed infiltration of cytotoxic and helper T cells in the tumour microenvironment upon ERV induction in mouse models of cancer [160, 164]. Treatment with DNMTis to induce viral mimicry enhances CD8^+^ T cell activation and detection of cytotoxic mediators *in vitro* [163, 165]. ERV expression may also contribute to an adaptive immune response through the production of tumour associated neoantigens (**Fig. 3c**) [166]. HERVs can produce epitopes presented as MHC-I bound peptides at the surface of tumour cells, which can increase tumour cell visibility to immune surveillance, resulting in a cytolytic T cell response [158, 167].**”**

*In Fig. 3, How does Type I/III interferons increase antigen presentation? The 2 arrows in the figure from "Type I & III interferons" to "antigen processing & presentation" made it seem like an intracellular pathway. However, it's more likely to involve autocrine and paracrine pathways. Type 1/3 interferons production -> release -> bind to IFN receptor on cell surface -> induce interferon stimulated genes (ISGs) -> ISGs increase antigen processing and presentation.*

We have removed the arrows, and changed the position of the MHC-I molecule to reflect an increase in ERV derived peptides that are being presented alongside increased IFN signalling. We have described ERV-derived tumour associated antigen presentation as a process that occurs in parallel with IFN induction in the figure legend, and have also elaborated on this further in the text. The figure legend now states “Viral mimicry synergises with production and presentation of ERV-derived tumour associated antigens (green dot) via MHC-I, to increase the visibility of tumour cells for immunogenic death by cytolytic T cell activation.” The relevant main text (page 12, line 10) now reads: “ERV expression may also contribute to an adaptive immune response through the production of tumour associated neoantigens (**Fig. 3c**) [166]. HERVs can produce epitopes presented as MHC-I bound peptides at the surface of tumour cells, which can increase tumour cell visibility to immune surveillance, resulting in a cytolytic T cell response [158,167].”

*One of the highlights of this review is to point out whether it is ERVs or Alus that triggers viral mimicry upon epigenetic therapy. This is an important point, and authors should be more comprehensive in reviewing literature (e.g. Ohtani et al., Cancer Research 2020, Activation of a Subset of Evolutionarily Young Transposable Elements and Innate Immunity Are Linked to Clinical Responses to 5-Azacytidine) and in proposing strategies so that the mechanism can be elucidated.*

We have expanded this section (starting page 12, line 39) to include the suggested literature, as well as a number of other studies that suggest other TEs are capable of inducing viral mimicry in cancer. It now reads: “ERV demethylation and transcription as a trigger for innate immune activation is proposed to explain the clinical impact of DNMTi treatment [53, 127, 128]. While an exciting and worthwhile concept, we consider this position with some caution, and doubly so considering the clinical implications. Moving the focus away from ERVs, multiple studies have tied viral mimicry to LINEs and SINEs [159]. In colorectal cancer cells, viral mimicry has been primarily associated with inverted *Alu* copy dsRNAs [174], whereas in a DNMTi-treated glioblastoma cell line, SVA and ERV upregulation are comparable [175]. In haematological datasets, expression from a diverse array of TEs, including ERVs, SINEs and LINEs, has been linked with viral mimicry upon DNMTi treatment [176]. Inactivation of the HUSH complex component MPP8 leads to both ERV and L1 upregulation, with the latter associated with dsRNA production and a type I interferon response [177]. Global ERV expression levels have been found to not predict clinical response of myelodysplasias treated with DNMTis [178]. Taken together, these results suggest that the ERV-centric model of viral mimicry upon treatment with DNMTis may be too simplistic. There is a pressing need, in our view, to move beyond inference of viral mimicry from correlations with TE family transcript levels. One approach could involve ectopic expression of various TEs upregulated in DNMTi-responsive patients, as shown to yield

a gene expression signature consistent with innate immune activation [176, 177]. Arguably the most direct evidence for TE-induced viral mimicry to date comes from an RNA protection assay for MDA5 in DNMTi-treated colorectal cancer cells [174]. Here, *Alu*, and not ERV or L1, transcripts were significantly enriched in MDA5-protected RNA, which argues that *Alu*s are the main source of immunogenic RNA in this model [174]. Such experiments expand the viral mimicry model to include non-ERV TEs as potential therapeutic targets in cancer [159, 174, 177]. Further experiments are required to resolve the individual TE copies that are capable of inducing viral mimicry, and directly test their immunogenicity.

*One of the biggest challenges in studying and targeting ERVs is related to the specificity. Numerous studies examined ERVs at family or subfamily level, whereas a few were able to target specific loci. This point should be made clearer with examples. Importantly, this is a future direction worth expanding. With emerging technologies, including long-read sequencing and genome / epigenome editing, more specifically, what kinds of future investigations can be possible?*

We agreed that this was an important point, and one of the main purposes of our review. We had mentioned long-read sequencing and CRISPR-dCas9 approaches in the earlier version of the text, but considering this and other suggestions from Reviewer #2 we added a new paragraph (page 13, line 50) stating: “Given recent evidence implicating a number of TE families in triggering viral mimicry, it would now be beneficial to directly dissect and modulate ERV activity in preclinical tumour models. A challenge in targeting ERVs, and TEs in general, is specificity; most studies of ERV function in cancer have examined entire ERV families, whereas few have targeted specific loci [41, 127, 128, 160]. Data obtained from cancer cell lines have shown that *in vitro* transcribed ERVs can trigger gene expression changes that indicate viral mimicry, and that different ERVs evoke different gene expression signatures [176]. This highlights the need to dissect ERV families and individual ERV loci in mechanistic explanations founded on viral mimicry. To be more clinically relevant, these studies could be expanded to study the modulation of ERVs and other endogenous TEs *in situ* via CRISPR-Cas9 and -dCas9 strategies [41]. As CRISPR-Cas9 genome editing relies upon guide RNA (gRNA) identity to specific target sequences, and ERVs form families of closely related sequences, it is possible to CRISPR-Cas9 edit entire ERV families, as well as individual ERV loci [9, 11, 15, 41, 91]. In addition to providing a deeper and more precise understanding of ERV loci that could be immunogenic, this approach could indicate more examples involved in onco-exaptation, such as the *APOC1* LTR2 enhancer highlighted by Deniz *et al.* [41]. CRISPR-dCas9 fusion proteins can also be employed to bring activating (CRISPRa) and interfering repressor (CRISPRi) complexes to specific TE loci. This approach has proven particularly useful for ERVs, where activator (e.g. CRISPR-dCas9-VPR) and repressor (e.g. CRISPR-dCas9-KRAB) fusion proteins can be used to assess the downstream effects of modulating ERV activity [9, 11, 41, 91, 92]. In the context of cancer, the downstream consequences for dsRNA production and interferon signalling upon ERV activation could then be assessed. Even if various obstacles to their application to cancer still need to be addressed, the use of CRISPR-Cas9 tools will greatly facilitate hypothesis testing surrounding ERV regulation and function, particularly in instances inferred from epigenome data and correlated transcription [19, 20, 39, 89, 90].”

*Fig 2 needs y-axis scale for genome browser views if the real data were used.*

We have added the y-axis scales and labels from the UCSC Genome Browser views of the

relevant data tracks shown in Figure 2.
